# Busulphan is active against neuroblastoma and medulloblastoma xenografts in athymic mice at clinically achievable plasma drug concentrations

**DOI:** 10.1038/sj.bjc.6690126

**Published:** 1999-02

**Authors:** I Boland, G Vassal, J Morizet, M-J Terrier-Lacombe, D Valteau-Couanet, C Kalifa, O Hartmann, A Gouyette

**Affiliations:** Laboratory of Pharmacotoxicology and Pharmacogenetics (CNRS URA147), Institut Gustave-Roussy, 39 rue Camille Desmoulins, Villejuif cedex, 94805, France; Department of Pediatric Oncology, Institut Gustave-Roussy, 39 rue Camille Desmoulins, Villejuif cedex, 94805, France; Department of Anatomopathology, Institut Gustave-Roussy, 39 rue Camille Desmoulins, Villejuif cedex, 94805, France

**Keywords:** brain tumours, childhood cancer, bone marrow transplantation, pharmacokinetics

## Abstract

High-dose busulphan-containing chemotherapy regimens have shown high response rates in children with relapsed or refractory neuroblastoma, Ewing's sarcoma and medulloblastoma. However, the anti-tumour activity of busulfan as a single agent remains to be defined, and this was evaluated in athymic mice bearing advanced stage subcutaneous paediatric solid tumour xenografts. Because busulphan is highly insoluble in water, the use of several vehicles for enteral and parenteral administration was first investigated in terms of pharmacokinetics and toxicity. The highest bioavailability was obtained with busulphan in DMSO administered i.p. When busulphan was suspended in carboxymethylcellulose and given orally or i.p., the bioavailability was poor. Then, in the therapeutic experiments, busulphan in DMSO was administered i.p. on days 0 and 4. At the maximum tolerated total dose (50 mg kg^−1^), busulphan induced a significant tumour growth delay, ranging from 12 to 34 days in the three neuroblastomas evaluated and in one out of three medulloblastomas. At a dose level above the maximum tolerated dose, busulphan induced complete and partial tumour regressions. Busulphan was inactive in a peripheral primitive neuroectodermal tumour (PNET) xenograft. When busulphan pharmacokinetics in mice and humans were considered, the estimated systemic exposure at the therapeutically active dose in mice (113 μg h ml^−1^) was close to the mean total systemic exposure in children receiving high-dose busulphan (102.4 μg h ml^−1^). In conclusion, busulphan displayed a significant anti-tumour activity in neuroblastoma and medulloblastoma xenografts at plasma drug concentrations which can be achieved clinically in children receiving high-dose busulphan-containing regimens. *1999 Cancer Research Campaign*


*British Journal of Cancer* (1999) **79**(5/6), 787–792
© 1999 Cancer Research Campaign
Article no. bjoc.1998.0126

### Busulphan is active against neuroblastoma and medulloblastoma xenografts in athymic mice at clinically achievable plasma drug concentrations

**I Boland[1], G Vassal[1,2], J Morizet[1], M-J Terrier-Lacombe[3], D Valteau-Couanet[2], C Kalifa[2], O Hartmann[2] and A Gouyette[1]**

[1]Laboratory of Pharmacotoxicology and Pharmacogenetics (CNRS URA147), [2]Department of Pediatric Oncology, [3]Department of Anatomopathology, Institut Gustave-Roussy, 39 rue Camille Desmoulins, 94805 Villejuif cedex, France

**Summary** High-dose busulphan-containing chemotherapy regimens have shown high response rates in children with relapsed or refractory neuroblastoma, Ewing's sarcoma and medulloblastoma. However, the anti-tumour activity of busulfan as a single agent remains to be defined, and this was evaluated in athymic mice bearing advanced stage subcutaneous paediatric solid tumour xenografts. Because busulphan is highly insoluble in water, the use of several vehicles for enteral and parenteral administration was first investigated in terms of pharmacokinetics and toxicity. The highest bioavailability was obtained with busulphan in DMSO administered i.p. When busulphan was suspended in carboxymethylcellulose and given orally or i.p., the bioavailability was poor. Then, in the therapeutic experiments, busulphan in DMSO was administered i.p. on days 0 and 4. At the maximum tolerated total dose (50 mg $kg^{-1}$), busulphan induced a significant tumour growth delay, ranging from 12 to 34 days in the three neuroblastomas evaluated and in one out of three medulloblastomas. At a dose level above the maximum tolerated dose, busulphan induced complete and partial tumour regressions. Busulphan was inactive in a peripheral primitive neuroectodermal tumour (PNET) xenograft. When busulphan pharmacokinetics in mice and humans were considered, the estimated systemic exposure at the therapeutically active dose in mice (113 μg h $ml^{-1}$) was close to the mean total systemic exposure in children receiving high-dose busulphan (102.4 μg h $ml^{-1}$). In conclusion, busulphan displayed a significant anti-tumour activity in neuroblastoma and medulloblastoma xenografts at plasma drug concentrations which can be achieved clinically in children receiving high-dose busulphan-containing regimens.

**Keywords:** brain tumours; childhood cancer; bone marrow transplantation; pharmacokinetics

Busulphan is an alkylating agent widely used in high-dose combined chemotherapy regimens before allogeneic or autologous bone marrow stem cells transplantation. Busulphan is cytotoxic to quiescent cells, especially to bone marrow stem cells (Dunn 1974). High-dose busulphan is used either as an alternative to total body irradiation, particularly in children undergoing allogeneic bone marrow transplantation for genetic diseases, or as an anti-cancer drug against haematological diseases such as acute and chronic myeloid leukaemias.

For a long time, busulphan was considered inactive in malignant solid tumours, as shown in the early clinical studies conducted in the late 1960s with long-lasting administration schedules (Sullivan, 1958; Arduino et al, 1967). However, the specific cellular target of busulphan, i.e. non-dividing cells, made this drug particularly attractive for the treatment of solid tumours because of its potential capacity to kill quiescent tumour cells while residual disease is minimal. Based on this hypothesis, we designed several high-dose busulphan-containing regimens in combination with other alkylating agents, i.e. cyclophosphamide, melphalan or thiotepa, for the treatment of paediatric malignant solid tumours. The clinical phase II evaluation of these combined regimens in relapsed or refractory diseases indicated that high response rates could be achieved in neuroblastomas (Hartmann et al, 1986) and Ewing's sarcomas and medulloblastomas (Kalifa et al, 1992; Dupuis-Girod et al, 1996). In addition, the use of a high-dose busulphan containing regimen was shown to be a strong favourable prognostic factor in terms of disease-free survival in patients with metastatic neuroblastoma (Hartmann et al, 1997) and Ewing's sarcoma (Ladenstein et al, 1997).

However, an important question remains unanswered: is busulphan active as a single agent against paediatric solid tumours? Because tumour xenografts are good preclinical models to evaluate the therapeutic activity of anti-cancer drugs (Mattern et al, 1988), we addressed this issue by studying busulphan therapeutic activity against paediatric malignant tumour xenografts, along with busulphan pharmacokinetics in nude mice.

*Received 9 January 1998*
*Revised 15 May 1998*
*Accepted 7 July 1998*

*Correspondence to:* G Vassal, Institut Gustave Roussy, 39, Rue C. Desmoulins, 94805 Villejuif cedex, France

### MATERIALS AND METHODS

### Drug

Busulphan was purchased from Sigma (France). Busulphan crystals were first dissolved in acetone, and a fine white powder was obtained after spontaneous evaporation.

### Animals

Female SPF-Swiss athymic mice were bred in the Animal Experimentation Unit, at the Institut Gustave-Roussy (Villejuif,

France). The strain was obtained from Carl Hansen (NIH, Bethesda, MD, USA) in 1976. Animals were housed in sterile isolators, and fed with irradiated nutriments (UAR, Villemoisson, Orge, France) and filtered water ad libitum. Experiments were carried out under the conditions established by the European Community (directive no. 86/609/CEE).

### Xenografts

The panel comprised one peripheral primitive neuroectodermal tumour (PNET) exhibiting the EWS-FLI fusion transcript (SKNMC), three neuroblastomas (IGRN835, IGRNB3, IGRNB8) and three medulloblastomas (IGRM21, IGRM33, IGRM34). These xenografts were derived either from established in vitro cell lines (SKNMC, IGRN835) or from primary tumours by subcutaneous transplantation of small fragments in previously irradiated athymic mice (Vassal et al, 1996 *a*). The SKNMC cell line was purchased from the American Type Culture Collection. The IGRN835 cell line was established in vitro by J Bénard from a previously treated stage IV neuroblastoma (Bettan-Renaud et al, 1989). IGRNB3 and IGRNB8 xenografts were established from newly diagnosed stage III neuroblastomas. These three xenografts exhibit several molecular and cytogenetic markers of poor prognosis neuroblastoma in children (Vassal et al, 1996 *b*). IGRM21 was derived from a newly diagnosed undifferentiated medulloblastoma, and IGRM33 from a recurrent medulloblastoma after conventional chemotherapy and radiotherapy. IGRM34 was derived from a metastatic PNET of the posterior fossa in a 1.6-year-old boy, and exhibited the histological features of a PNET with a rhabdoid phenotype. These three medulloblastoma xenografts have been previously described and characterized (Vassal et al, 1996 *a*, 1997). The human origin of the xenografts was confirmed by the presence of human lactate dehydrogenase (LDH) isoenzymes and by cytogenetic analysis.

All the xenografts were maintained in vivo by sequential passaging from s.c. implants, with an engraftment success rate exceeding 75%. The stability of the xenografts was checked at each passage by analysing the doubling time, and every 3–6 passages by histological analysis. Drug treatment experiments were carried out between passages 6 and 17.

### Drug formulation and pharmacokinetics

To evaluate the optimal vehicle and route for the in vivo administration of busulphan, plasma pharmacokinetics and toxicity were first evaluated after oral, intraperitoneal and intravenous administration of busulphan in different preparations: a solution in DMSO (Sigma), a suspension in 2.5% carboxymethylcellulose (CMC) (Aldrich, France) in water, or a suspension in a mixture of CMC and DMSO. For this last preparation, busulphan was first dissolved in DMSO (24 mg in 0.4 ml) and then suspended in 2.5% CMC in water.

For the pharmacokinetic analysis, tumour-free animals aged 7–8 weeks received a single dose of 40 mg kg$^{-1}$ prepared in one of the different vehicles via one of the three routes of administration. Three animals were sacrificed by carbon dioxide inhalation before and after the administration at each of the following time points: 15, 30 min, 1, 2, 3, 5 and 7 h. A total blood sample (1 ml) was immediately collected by cardiac puncture in a heparinized vial and plasma was separated by centrifugation. At each time point, plasma samples from the three animals were pooled and frozen at –80°C until analysis.

Plasma busulphan levels were measured by gas chromatography mass spectrometry, using a tetradeuterated internal standard as previously described (Vassal et al, 1988).

Model-independent pharmacokinetic analysis was performed using the WinNonlin program (Scientific Consulting, Cary, NC, USA). The decrease in the concentration–time curve was monoexponential. Elimination half-lives were determined by linear regression from the linear portion of the log curve. The areas under the curve (AUC) were calculated using the linear/log trapezoidal rule from 0 to 7 h, and extrapolated to infinity according to the elimination half-life. The total plasma clearance rates (CI) were calculated by dividing the dose by the AUC. The apparent volumes of distribution ($V_d$) were calculated by dividing the clearance rate by the slope of the linear portion of the log curve. For each route and vehicle, the relative bioavailability (*F*) was calculated by dividing the observed AUC by the AUC of the reference dosage, which proved to be i.p administration in DMSO.

The toxicity of each route and vehicle evaluated was studied in tumour-bearing animals at doses from 37 to 200 mg kg$^{-1}$, and expressed as the dose that killed 50% of the treated animals ($LD_{50}$).

### Experimental design

Drug activity against bilateral advanced stage tumours was evaluated as previously described (Vassal et al, 1996 *b*). For each experiment, tumour fragments (15 mm$^3$) were xenotransplanted subcutaneously in 30–50 athymic mice aged 6–8 weeks. On day 0 of the treatment, mice bearing a 100–400 mm$^3$ subcutaneous tumour were pooled and randomly assigned to 2–4 groups of 6–9 animals, including one control and 1–3 treated groups. Two tumour perpendicular diameters were measured three times weekly with a caliper by the same investigator. Each tumour volume was calculated according to the following equation: $V$ (mm$^3$) = $d^2$ (mm$^2$) × $D$ (mm)/2, where $d$ and $D$ are the smallest and largest perpendicular tumour diameters respectively. Each group of mice was treated according to the average weight of the group. Animal body weights were recorded three times weekly. Mortality was checked daily and an autopsy was performed when appropriate. Body weight loss (BWL) was reported as the maximum treatment-related body weight loss. The experiments lasted until tumour volumes reached 1500–2000 mm$^3$.

### Treatment

The objective was to evaluate busulphan anti-tumour activity at the maximum tolerated dose. Thus, two dose levels, including one toxic dose level, were used during each experiment. Busulphan in DMSO was administered i.p. at a dose of 25 and 31.2 mg kg$^{-1}$ (0.1 ml per animal) on days 0 and 4 for the evaluation of anti-tumour activity.

### Evaluation of anti-tumour activity

Busulphan therapeutic activity was evaluated according to two criteria: (i) the number of complete and partial tumour regressions; (ii) the tumour growth delay (TGD). The total tumour burden in each animal bearing bilateral tumours was considered for the evaluation of tumour regression. Complete regression (CR) was defined as a tumour regression beyond the palpable limit (15 mm$^3$), and partial regression (PR) as a tumour regression greater than 50% of the initial tumour volume. CR and PR had to be observed for at least two

© Cancer Research Campaign 1999

**Table 1** Plasma pharmacokinetics of busulphan (40 mg kg$^{-1}$) in athymic mice using different vehicles and routes of administration

| Vehicle | Route | $C_{max}$ (μg ml$^{-1}$) | $T_{1/2}$ (h) | $AUC_{0-\infty}$ (μg h ml$^{-1}$) | *F* | Cl (ml h$^{-1}$) | $V_d$ (ml) |
|---|---|---|---|---|---|---|---|
| CMC | p.o. | 2.8 | NA | 4.4 | 0.05 | 228 | NA |
| CMC | i.p. | 11.1 | NA | 28.9 | 0.31 | 34 | NA |
| CMC/DMSO | i.p. | 33 | 0.86 | 54.3 | 0.59 | 18 | 22.5 |
| DMSO | i.p. | 44.8 | 1.16 | 92.2 | 1 | 9.3 | 15.4 |
| DMSO | i.v. | 26.3 | 1.30 | 80 | 0.86 | 11 | 26 |
| DMSO | i.v. | 26.9 | 1.40 | 75.6 | 0.82 | 11.7 | 23.9 |

CMC, carboxymethylcellulose; DMSO, dimethylsulphoxide; p.o., per os; i.p., intraperitoneal; $C_{max}$, maximum plasma concentration; $T_{1/2}$, elimination half-life; $AUC_{0-\infty}$, area under the curve extrapolated to infinity; *F*, bioavailability; Cl, clearance rate; $V_d$, volume of distribution; $LD_{50}$, dose that kills 50% of animals; NA, not available. Pharmacokinetics after i.v. administration were evaluated in two separate experiments.

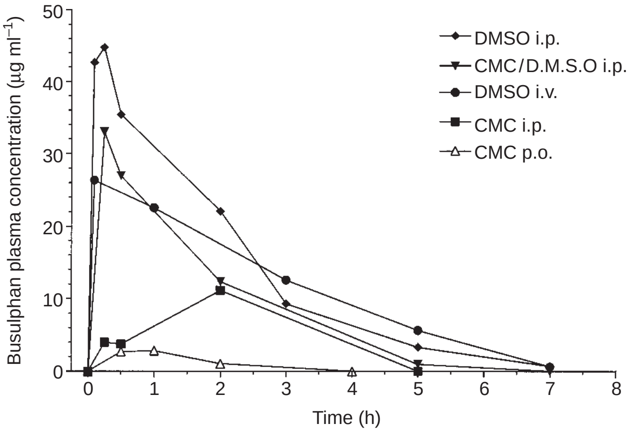

**Figure 1** Busulphan plasma disposition in athymic mice. CMC, carboxymethylcellulose; DMSO, dimethylsulphoxide; p.o., per os; i.p., intraperitoneal consecutive tumour measurements in order to be retained. The TGD was defined as the difference between the treated group and the control group in the median time to reach a tumour volume that was five times greater than the initial tumour volume (Vassal, 1996*b*).

### Statistical analysis

For each experiment, the times for each tumour to attain a volume five times greater than the initial tumour volume in the treated and control groups were compared using a two-sided non-parametric Mann–Whitney test.

### RESULTS

### Optimal drug formulation and route of administration

Because busulphan is highly hydrophobic, we first investigated the use of several vehicles for enteral and parenteral administration, in terms of pharmacokinetics and toxicity (Table 1). After a single administration of 40 mg kg$^{-1}$, the highest AUC was obtained with busulphan in DMSO administered i.p., and this was considered the reference preparation and route. When busulphan was suspended in carboxymethylcellulose, bioavailability (*F*) was poor after i.p. injection (*F* = 31%), and even poorer when given orally (*F* = 5%) (Figure 1A). With the latter formulation, the $LD_{50}$ could not be established. When busulphan was administered in a DMSO/CMC mixture, bioavailability was improved, up to 59%, and the $LD_{50}$ was 125 mg kg$^{-1}$. When busulphan was administered in DMSO, equivalent $LD_{50}$s, i.e. 60–65 mg kg$^{-1}$, were observed after i.p. and i.v. injection. Nevertheless, the bioavailability of the i.v. form was 86% compared with that of the i.p. injection (Figure 1B). This unexpected result was confirmed in a second separate experiment (Table 1). This difference was attributed to the venous toxicity of the formulation which induced leakages. In addition, local cutaneous tolerance after a single i.v. administration was poor. The i.p. administration of busulphan in DMSO was thus considered the optimal route and preparation in terms of bioavailability and local tolerance.

### Toxicity

During the seven therapeutic experiments, busulphan was given i.p. in DMSO on day 0 and day 4, at two total dose levels, i.e. 50 and 62.5 mg kg$^{-1}$. Overall, treatment-related deaths occurred in 4 out of 52 animals (7.6%) and 25 out of 52 animals (48%) at the 50 and 62.5 mg kg$^{-1}$ dose levels respectively. The dose of 50 mg kg$^{-1}$ was thus considered as the maximum tolerated dose. Treatment-related deaths were delayed, with a median day of onset on day 21 (range 15–24). The early body weight loss was not predictive of treatment-related death. The delayed treatment-related death allowed the evaluation of tumour regression at dose levels above the maximum tolerated dose. This is of interest when studying a compound which is used in the clinical setting at high doses with bone marrow stem cell rescue. Diffuse purpura and anaemia were the clinical manifestations of busulphan-induced toxicity. At higher dose levels, diarrhoea was also observed.

### Anti-tumour activity

At the maximum tolerated total dose of 50 mg kg$^{-1}$, busulphan induced a significant tumour growth delay ranging from 12 to 34 days in the three neuroblastoma xenografts evaluated (Table 2). IGRNB3 was the most sensitive, with two of seven animals showing partial regression of their total tumour burden. Complete and partial regressions were observed at a higher toxic dose in the other two neuroblastomas, IGRN835 and IGRNB8. Busulphan was significantly active in one of three medulloblastoma xenografts. Indeed, one complete regression with a tumour growth

© Cancer Research Campaign 1999 

**Table 2** Anti-tumour activity of i.p. busulphan in athymic mice bearing advanced stage subcutaneous paediatric solid tumour xenografts

| Xenograft (histology) | DT (day) | Daily dose ($mg\ kg^{-1}$) | Total dose ($mg\ kg^{-1}$) | *n* | BWL (%) | Toxic death | CR | PR | TGD (day) | *P*-value |
|---|---|---|---|---|---|---|---|---|---|---|
| IGRN835 | 4.5 | 25 | 50 | 7 | 2 | 0 | 0 | 0 | 12 | <0.01 |
| (NB) | | 31.25 | 62.5 | 8 | 0.5 | 3 | 1 | 2 | NE | |
| IGRNB8 | 4 | 25 | 50 | 7 | 0 | 0 | 0 | 0 | 13 | <0.0001 |
| (NB) | | 31.25 | 62.5 | 7 | 7 | 6 | 0 | 2 | NE | |
| IGRNB3 | 7 | 25 | 50 | 8 | 0 | 1 | 0 | 2 | 34 | <0.0001 |
| (NB) | | 31.25 | 62.5 | 8 | 0 | 6 | 0 | 1 | NE | |
| IGRM21 | 9 | 25 | 50 | 6 | 1 | 0 | 1 | 0 | 33 | <0.0001 |
| (MB) | | 31.25 | 62.5 | 6 | 5 | 1 | 0 | 5 | 65 | <0.0001 |
| IGRM33 | 15 | 25 | 50 | 8 | 0 | 1 | 0 | 0 | 0 | NS |
| (MB) | | 31.25 | 62.5 | 8 | 0 | 4 | 0 | 0 | NE | |
| IGRM34 | 3 | 25 | 50 | 7 | 5 | 1 | 0 | 0 | 6 | NS |
| (MB) | | 31.25 | 62.5 | 7 | 5 | 3 | 0 | 0 | NE | |
| SKNMC | 6 | 25 | 50 | 9 | 0 | 1 | 0 | 0 | 7 | NS |
| (pPNET) | | 31.25 | 62.5 | 9 | 0 | 2 | 0 | 0 | NE | |

DT, tumour doubling time; BWL, maximum body weight loss; CR, complete regression; PR, partial regression; TGD, tumour growth delay; NB, neuroblastoma; MB, medulloblastoma; pPNET, peripheral primitive neuroectodermal tumour; NA, not evaluable. For each experiment, the times of each tumour to attain a volume five times greater than the initial tumour volume in the treated and control groups were compared using a two-sided non-parametric Mann–Whitney test.

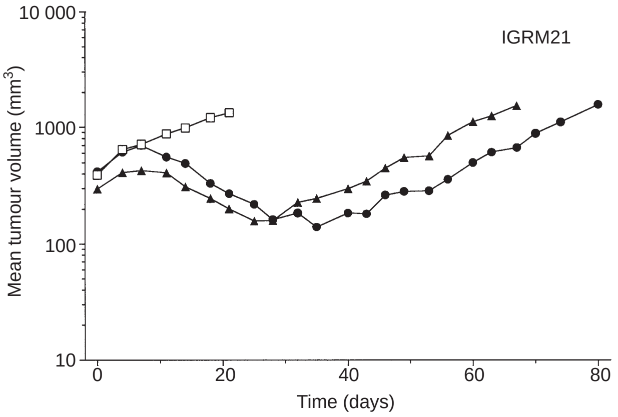

**Figure 2** Anti-tumour activity of busulphan against a medulloblastoma xenograft (IGRM21). Busulphan was given i.p. in DMSO at a dose of 25 $mg\ kg^{-1}$ (▲) and 31 $mg\ kg^{-1}$ (●) on days 0 and 4. Animals in the control group (□) received i.p. DMSO. Each line represents the evolution of the mean tumour volume in each group delay of 33 days was observed in IGRM21 at the 50 $mg\ kg^{-1}$ total dose level (Figure 2). At a higher dose level, five out of six partial regressions of the total tumour burden were observed in IGRM21-bearing animals, with a significant tumour growth delay of 65 days. No therapeutic activity was observed in the last two medulloblastoma and the peripheral PNET xenografts. Busulphan proved to be active in four out of seven paediatric solid tumour xenografts, and a dose–effect relationship was suggested by tumour regressions observed at dose levels above the maximum tolerated dose.

### DISCUSSION

Busulphan is an old drug, whose clinical development began in the early 1950s. The preclinical evaluation of its anti-tumour activity was limited until the recent development of structurally related compounds, such as hepsulfam (Berger et al, 1992). Busulphan has, however, been widely used for in vivo preclinical studies on haematopoiesis and allogeneic bone marrow transplantation (Josvasen et al, 1973; Tutschka et al, 1977).

Busulphan is poorly soluble in water. Until recently, only an oral form was available for the administration of conventional and high doses in humans. Several vehicles have, however, been reported for enteral and parenteral administration in animals, including corn oil (Josvasen et al, 1973; Millar et al, 1975), peanut oil (MacCracken et al, 1988), carboxymethylcellulose (Tutschka et al, 1977), DMSO (Smalowski et al, 1989), acetone and water (Bhoopalam et al, 1985; Down et al, 1989), and mixtures of these different vehicles. Aaron et al (1994) used a single i.p. injection of busulphan in 10% DMSO in saline and found a $DL_{10}$ of 60.3 $mg\ m^{-2}$ in athymic BALB/c mice bearing CNS tumour xenografts. Berger et al (1992) reported the use of i.p. administration of busulphan in pure DMSO in NMRI athymic mice, delivering an optimal dose of 150 $mg\ kg^{-1}$ in a single i.p. injection. We showed that the bioavailability and toxicity of busulphan was highly dependent on the vehicle and route of administration. When pharmacokinetics and local tolerance were considered, intraperitoneal administration of busulphan in DMSO was clearly the optimal route and formulation for in vivo preclinical studies. These pharmacological results might be of interest for the conduct of other in vivo preclinical studies, such as those evaluating gene-modified stem cell transplantation (Yeager et al, 1991). A new drug formulation in dimethylacetate and polyethyleneglycol has recently been developed for parenteral administration of busulphan in humans (Bhagwatwar et al, 1996).

The preclinical evaluation of the in vivo anti-tumour activity of busulphan has so far been limited in malignant solid tumour models. While comparing hepsulfam to busulphan, Berger et al (1992) showed that busulphan induced tumour growth delays without tumour regressions in two subcutaneous human tumour xenografts in athymic mice (stomach and lung carcinomas), and was inactive in a human melanoma xenograft. In addition, when busulphan activity was investigated in vitro against a panel of

 © Cancer Research Campaign 1999

different malignant solid tumour cell lines, busulphan was cytotoxic at the highest concentration of 10 μg $ml^{-1}$ in 12 out of 37 cell lines (32%), including gastric, lung, breast and testicular cancers and melanoma. Aaron et al (1994) addressed the issue of busulphan anti-tumour activity in brain tumour xenografts in athymic mice. Busulphan induced a significant tumour growth delay in one medulloblastoma, two ependymomas and four out of five high-grade gliomas subcutaneous xenografts evaluated at an advanced stage. In addition, busulphan increased the median survival of animals bearing intracranial brain tumour xenografts. Our study showed that busulphan displayed significant anti-tumour activity in vivo in three out of three advanced stage subcutaneous neuroblastoma xenografts, and in one of three medulloblastoma xenografts. This activity was dose dependent, as shown by the increased number of complete and partial regressions above the maximum tolerated dose. For a long time, busulphan cytotoxicity was considered to be restricted to haematological malignancies, and especially myeloid leukaemias. Altogether, these preclinical data demonstrate that single-agent busulphan displays anti-tumour activity against malignant solid tumours of various histological types and even complete regression of some of these tumours. High-dose busulphan-containing regimens are currently being investigated for the treatment of advanced and metastatic breast carcinomas (Demirer et al, 1996).

For the first time, we report the significant in vivo anti-tumour activity of busulphan against three of three neuroblastoma xenografts exhibiting molecular markers of poor prognosis in children. Metastatic neuroblastoma is a frequent malignant solid tumour in children. Intensified chemotherapy with high-dose regimens and bone marrow stem cell support have succeeded in increasing the response rate in refractory or relapsed metastatic neuroblastomas. During the last 15 years, we have developed high-dose busulphan-containing regimens for the treatment of metastatic neuroblastoma (Hartmann et al, 1986). Recently, in a multivariate retrospective analysis of 218 patients over 1 year of age treated in a single centre with high-dose chemotherapy and haematopoietic stem cell transplantation, we showed that the presence of the busulphan–melphalan combination in the regimen was a strong independent favourable prognostic factor (Hartmann et al, 1997).

From a pharmacological point of view, busulphan is a very attractive drug for the treatment of central nervous system malignant diseases. After oral administration, it is widely distributed in the cerebrospinal fluid (CSF) with a mean CSF to plasma ratio of 1 (Vassal et al, 1990). Hassan et al (1992) studied brain tissue distribution of [$^{11}$C]busulphan using positron-emission tomography scans in non-human primates and humans (Hassan et al, 1992). This study showed that 20% of the drug was rapidly and homogeneously distributed throughout the brain tissue and mainly in the white matter. Our preclinical study showed that busulphan was very active in one of three medulloblastoma xenografts. These results along with those reported by Aaron et al (1994) in several different brain tumour xenografts suggest that busulphan may be an important drug worth considering for the treatment of brain tumours in children. Using a busulphan–thiotepa regimen, we have already reported a 40–75% response rate in children with relapsed or refractory medulloblastoma (Kalifa et al, 1992; Dupuis-Girod et al, 1996).

Finally, the pharmacokinetic study performed to determine of the optimal vehicle and route of administration for busulphan in animals allowed us to compare the busulphan disposition in nude mice and humans. The elimination half-life was shorter in mice (0.86–1.4 h) than in humans (2–3 h). The total plasma clearance rate was 9.3 ml $h^{-1}$ in mice receiving a single i.p. administration of 40 mg $kg^{-1}$. Assuming linear pharmacokinetics, the systemic exposure obtained in mice at the maximum tolerated total dose, i.e. 50 mg $kg^{-1}$, was expected to be 113 μg h $ml^{-1}$. We have developed a new oral dosage for children, i.e. 600 mg $m^{-2}$, which is able to eliminate the effect of age on busulphan plasma clearance in children (Vassal et al, 1992). In this regimen, busulphan is given orally at a dose of 37.5 mg $m^{-2}$ every 6 h for 16 consecutive doses. The mean systemic exposure after the first dose of this regimen was 6.4 μg h $ml^{-1}$ in 25 children aged 2–14 years. It can be extrapolated that the mean total systemic exposure during the 4-day treatment would be 102.4 μg h $ml^{-1}$, regardless of the already described nychthemeral variations (Vassal et al, 1993). The therapeutic efficacy of busulphan in paediatric solid tumour xenografts in nude mice was therefore observed at total systemic exposure levels that can be regularly achieved in children receiving high-dose busulphan in combined chemotherapy regimens before autologous haematopoietic stem cell transplantation.

In conclusion, busulphan is an active alkylating agent against paediatric solid tumour xenografts at plasma levels that can be achieved clinically in children receiving a high-dose busulphan-containing regimen. These preclinical results suggest that busulphan may be an active drug when used at high dose in combination with another alkylating agent in childhood solid tumours, and especially in neuroblastoma and medulloblastoma at a stage of minimal residual disease.

### ACKNOWLEDGEMENTS

The authors thank Patrice Ardouin and all the staff of the Animal Experimentation Unit, Institut Gustave-Roussy, for the care of animals, and Mrs L Saint-Ange for editing the manuscript. This work was supported in part by grants from the Ligue Nationale de Lutte contre le Cancer.

### REFERENCES

Aaron RH, Elion GB, Colvin OM, Graham M, Keir S, Bigner DD and Friedman HS (1994) Busulfan therapy of central nervous system xenografts in athymic mice. *Cancer Chemother Pharmacol* **35**: 127–131

Arduino LJ and Mellinger GT (1967) Clinical trial of busulfan (NSC-750) in advanced carcinoma of prostate. *Cancer Chemother Rep* **51**: 295–304

Berger DP, Winterhalter BR, Dengler WA and Fiebig HH (1992) Preclinical activity of hepsulfam and busulfan in solid human tumor xenografts and human bone marrow. *Anticancer Drugs* **3**: 531–539

Bettan-Renaud L, Bayle C, Teyssier JR and Benard J (1989) Stability of phenotypic and genotypic traits during the establishment of a human neuroblastoma cell line, IGR-N-835. *Int J Cancer* **44**: 460–466

Bhagwatwar HP, Phadungpojna S, Chow DSL and Anderson BS (1996) Formulation and stability of busulfan for intravenous administration in high-dose chemotherapy. *Cancer Chemother Pharmacol* **37**: 401–408

Bhoopalam N, Price KS, Norgello H and Fried W (1985) Busulfan-induced suppression of natural killer cell activity. *Exp Hematol* **13**: 1127–1132

Bishop JB and Wassom JS (1986) Toxicological review of busulfan (Myleran). *Mutat Res* **168**: 15–45

Demirer T, Buckner CD, Appelbaum FR, Clift R, Strob R, Myerson D, Lilleby K, Rowley S and Bensinger WI (1996) High-dose busulfan and cyclophosphamide followed by autologous transplantation in patients with advanced breast cancer. *Bone Marrow Transplant* **17**: 769–774

Down JD, Berman AJ, Warhol M, Van Dijken PJ, Ferrara JLM, Yeap B, Hellman S and Mauch PM (1989) Late tissue-specific toxicity of total body irradiation and busulfan in a murine bone marrow transplant model. *Int J Radiat Oncol Biol Phys* **17**: 109–116

Dunn CR (1974) The chemical and biological properties of busulphan (Myleran). *Exp Hematol* **2**: 101–117

© Cancer Research Campaign 1999 

Dupuis-Girod S, Hartmann O, Benhamou E, Doz F, Mechinaud F, Bouffet E, Coze C and Kalifa C (1996) Will high-dose chemotherapy followed by autologous bone marrow transplantation supplant cranio-spinal irradiation in young children treated for medulloblastoma? *J Neurooncol* **25**: 87–89

Hartmann O, Benhamou E, Beaujean F, Pico JL, Kalifa C, Patte C, Flamant F and Lemerle J (1986) High-dose busulfan and cyclophosphamide with autologous bone marrow transplantation support in advanced malignancies in children: a phase II study. *J Clin Oncol* **4**: 1804–1810

Hartmann O, Valteau-Couanet D, Lapierre V, Couanet D, Lumbroso J and Benhamou E (1997) Stage IV neuroblastoma over 1 year of age autografted: the combination used in the conditioning regimen is the major prognostic factor. *Med Ped Oncol* **20**: 320

Hassan M, Öberg G, Ericson K, Ehrsson H, Eriksson L, Ingvar M, Stone-Elander S, Thorell J-O, Smedmyr B, Warne N, and Widen L (1992) In vivo distribution of [$^{11}$C]-busulfan in cynomolgus monkey and in the brain of a human patient. *Cancer Chemother Pharmacol* **30**: 81–85

Josvasen N and Boyum A (1973) Haematopoiesis in busulphan-treated mice. A comparison between two different stem cell assays. *Scand J Haematol* **11**: 78–86

Kalifa C, Hartmann O, Demeocq F, Vassal G, Couanet D, Terrier-Lacombe MJ, Valteau D, Brugieres L and Lemerle J (1992) High-dose busulfan and thiotepa with autologous bone marrow transplantation in childhood malignant brain tumors: a phase II study. *Bone Marrow Transplant* **9**: 227–233

Ladenstein R, Hartmann O, Pinkerton R, Michon J, Garaventa A, Rosti G, Philip T (1997) The impact of megatherapy followed by stem cell reinfusion in Ewing tumour patients with residual disease. *Bone Marrow Transplant* **19**: S86

MacCracken III CH, Lottsfeldt JL and Lee MY (1988) Concurrent activation of granulocytes and osteoclasts in busulfan-suppressed bone marrow in response to transplantation of a mammary carcinoma in mice. *Exp Hematol* **16**: 285–288

Mattern J, Bak M, Hahn EW and Volm M (1988). Human tumor xenografts as a model for drug testing. *Cancer Metastasis Rev* **7**: 263–284

Millar JL, Hudspith BN and Blackett NM (1975) Reduced lethality in mice receiving a combined dose of cyclophosphamide and busulphan. *Br J Cancer* **32**: 193–198

Smalowski WE, Araneo BA, Butler MO, Fung MC and Jonhson HM (1989) Peripheral lymph node helper T-cell recovery after syngeneic bone marrow transplantation in mice prepared with either γ-irradiation or busulfan. *Blood* **74**: 1436–1445

Sullivan R (1958) Myleran therapy in bronchogenic carcinoma. *Ann NY Acad Sci* **68**: 1038–1046

Tutschka PJ and Santos GW (1977) Bone marrow transplantation in the busulfan-treated rat. III. Relationship between myelosuppression and immunosuppression for conditioning bone marrow recipients. *Transplantation* **24**: 52–62

Vassal G, Re M and Gouyette A (1988) Gas chromatographic–mass spectometric assay for busulfan in biological fluids using a deuterated internal standard. *J Chromatog* **428**: 357–361

Vassal G, Deroussent A, Hartmann O, Challine D, Benhamou E, Valteau-Couanet D, Brugieres L, Kalifa C, Gouyette A and Lemerle J (1990) Dose-dependent neurotoxicity of high-dose busulfan in children: a clinical and pharmacological study. *Cancer Res* **50**: 6203–6207

Vassal G, Deroussent A, Challine D, Hartmann O, Koscielny S, Valteau-Couanet D, Lemerle J and Gouyette A (1992). Is 600 mg/m$^2$ the appropriate dosage of busulfan in children undergoing bone marrow transplantation? *Blood* **79**: 2475–2479

Vassal G, Challine D, Koscielny S, Hartmann O, Deroussent A, Valteau-Couanet D, Lemerle J, Levi F and Gouyette A (1993) Chronopharmacology of high-dose busulfan in children. *Cancer Res* **53**: 1534–1537

Vassal G, Terrier-Lacombe MJ, Lellouch-Tubiana A, Valery CA, Sainte-Rose C, Morizet J, Ardouin P, Riou G, Kalifa C and Gouyette A (1996*a*) Tumorigenicity of cerebellar primitive neuro-ectodermal tumors in athymic mice correlates with poor prognosis in children. *Int J Cancer (Pediatr Oncol)* **69**: 146–151

Vassal G, Terrier-Lacombe MJ, Bissery MC, Venuat AM, Gyergyay F, Benard J, Morizet J, Boland I, Ardouin P, Bressac-De-Paillerets B and Gouyette A (1996*b*) Therapeutic activity of CPT-11, a DNA-topoisomerase I inhibitor, against peripheral primitive neuroectodermal tumour and neuroblastoma xenografts. *Br J Cancer* **74**: 537–545

Vassal G, Boland I, Santos A, Bissery MC, Terrier-Lacombe MJ, Morizet J, Sainte-Rose C, Lellouch-Tubiana A, Kalifa C and Gouyette A (1997) Potent therapeutic activity of irinotecan (CPT-11) and its schedule-dependency in medulloblastoma xenografts in nude mice. *Int J Cancer* **73**: 156–163

Yeager AM, Shinohara M and Shinn C (1991) Hematopoietic cell transplantation after administration of high-dose busulfan in murine globoid cell leukodystrophy (the Twitcher mouse). *Pediatr Res* **29**: 302–305

*© Cancer Research Campaign 1999*
